# Satellite Image Compression Guided by Regions of Interest

**DOI:** 10.3390/s23020730

**Published:** 2023-01-09

**Authors:** Christofer Schwartz, Ingo Sander, Fredrik Bruhn, Mathias Persson, Joakim Ekblad, Christer Fuglesang

**Affiliations:** 1 KTH Royal Institute of Technology, 100 44 Stockholm, Sweden; 2 Unibap AB, Kungsängsgatan 12, 753 22 Uppsala, Sweden; 3 School of Innovation, Design and Engineering (IDT), Embedded Systems Division, Mälardalen University, P.O. Box 883, 721 23 Västerås, Sweden; 4 Saab AB, Olof Palmes Gata 17, 111 22 Stockholm, Sweden

**Keywords:** satellite communication, image compression, cloud detection, vessel detection, change detection

## Abstract

Small satellites empower different applications for an affordable price. By dealing with a limited capacity for using instruments with high power consumption or high data-rate requirements, small satellite missions usually focus on specific monitoring and observation tasks. Considering that multispectral and hyperspectral sensors generate a significant amount of data subjected to communication channel impairments, bandwidth constraint is an important challenge in data transmission. That issue is addressed mainly by source and channel coding techniques aiming at an effective transmission. This paper targets a significant further bandwidth reduction by proposing an on-the-fly analysis on the satellite to decide which information is effectively useful before coding and transmitting. The images are tiled and classified using a set of detection algorithms after defining the least relevant content for general remote sensing applications. The methodology makes use of the red-band, green-band, blue-band, and near-infrared-band measurements to perform the classification of the content by managing a cloud detection algorithm, a change detection algorithm, and a vessel detection algorithm. Experiments for a set of typical scenarios of summer and winter days in Stockholm, Sweden, were conducted, and the results show that non-important content can be identified and discarded without compromising the predefined useful information for water and dry-land regions. For the evaluated images, only 22.3% of the information would need to be transmitted to the ground station to ensure the acquisition of all the important content, which illustrates the merits of the proposed method. Furthermore, the embedded platform’s constraints regarding processing time were analyzed by running the detection algorithms on Unibap’s iX10-100 space cloud platform.

## 1. Introduction

The number of satellites in orbit is growing fast. According to [1], the number of satellites in orbit in constellations for commercial purposes was around 4000 in 2022 and will be the double by 2024. Most satellite constellation projects are designed aiming at medium Earth orbit (MEO) and low Earth orbit (LEO) constellations. Among the target fields, 4% are related to space observations, 46% to earth observations, and 50% to communications. In case of satellites for earth observations, constellations are able to image the entire land surface of the Earth every day. Consequently, medium-resolution or high-resolution multispectral sensors (e.g., 4 bands or 8 bands) can generate a large amount of information daily. For hyperspectral sensors, the amount of data generated per area can be even greater, increasing the demand for bandwidth availability for data transmission.

In this context, image compression is an important strategy adopted by most communication systems, to reduce the bandwidth used in image transmission [2,3]. For aerospace applications, some algorithms have been recommended by the Consultative Committee for Space Data System (CCSDS). The CCSDS 122.0-B-2 Recommended Standard details an image compression algorithm that can be seen as a minimalist version of JPEG2000, in which a careful trade-off between compression performance and complexity has been made to make the compressor more suitable to be implemented in either hardware or software [4]. An alternative to the CCSDS image compressor is the JPEG-LS standard, presenting lower complexity and similar performance in terms of data compression, but this is limited to lossless and near-lossless compression [5]. Other general-purpose wavelet-based image compression algorithms capable of providing effective lossless and lossy compression can be found in [6,7]. Video compression methods focused on aerospace applications can also be found [8,9], aiming at eliminating long-term redundancy among multiple periodically revisited videos.

The images acquired by the satellite are compressed and transmitted to the ground-station assuming that: (1) all the images are equally important; and that (2) all the content in each image is equally important too. However, both assumptions are not true for some applications. As mentioned in [10], one of the limitations of small satellite missions is the platform’s capacity for using instruments with high power consumption or high data rate requirements. Thus, small satellite missions usually focus on one specific physical phenomenon to be observed and monitored. One can consider as small satellite projects constellations of pico-satellites, nano-satellites, and some of micro-satellites, which are common among constellation projects. In fact, among the satellites constellation projects accounted for in [1], 1% correspond to pico-satellites (mass less than 1 kg), 30% to nano-satellites (mass between 1 and 10 kg), and 18% to micro-satellites (mass between 10 and 100 kg). These percentages could be even greater, since 35% of constellation projects do not give information about the sizes of their satellites.

Overall, some acquired images can be considered useless, or partially useless, for some applications. For instance, cloud-covered images may not be useful for applications such as surveillance or deforestation mapping, and as a consequence, there is no need to transmit them to the ground-station. The potential of data saving for such applications is high, since the global cloud coverage (cloud fraction) is approximately 67%, according to information retrieved from the Moderate Resolution Imaging Spectroradiometer (MODIS) [11]. More precisely, MODIS detected that the cloud fraction over land is around 55%, with a distinctive seasonal cycle, whereas the ocean’s cloudiness is around 72%, and it has far less seasonal variation. For oceanic areas, even more images can be considered not useful if the application relies on vessel detection, which fits, for instance, Maritime Domain Awareness (MDA) applications. The potential of data saving for applications where other content than vessels can be discarded is huge, since the oceans cover 70% of the earth’s surface.

The previous knowledge about which content is not useful allows optimizing the image compression step for further information saving. In that sense, regions of interest (ROI) can be defined by deciding on-the-fly if the content of the images (or part of the content) is actually useful for the target application. Thus, the ROI can be compressed with higher compression rates than the other regions, preserving the image quality for the useful content while ensuring data savings from the others.

This paper targets significant data savings by proposing an on-the-fly analysis to guide the compression of images on satellites before transmission. Thus, additional bandwidth reduction can be achieved. The proposed method performs the detection of content having available measurements of the red band, green band, blue band (i.e., the RGB bands), and the near-infrared band (NIR band). The challenge is to achieve a sufficient probability of detection (PD) while keeping false alarm ratio (FAR) as low as possible, but within the expected platform’s constraints with respect to processing time and energy expenditure.

Preliminary results illustrating achievable gains in terms of information saved are described in [12]. In [12], information is discarded that is considered not useful content: (1) images covered by clouds and (2) water images without vessels. Thus, the methodology described in [12] is composed of a cloud detection algorithm and a vessel detection algorithm, and was evaluated using four satellite images.

In our study, the proposed method is based on three detection algorithms: a cloud detection algorithm, a change detection algorithm, and a vessel detection algorithm. In turn, better gains in dry-land regions can be achieved, ensuring the image quality when something interesting has significantly changed in an area or when a new type of object appears. Experiments using 25 satellite images were conducted, representing typical scenarios of summer and winter days in Stockholm, Sweden. Precisely, the behavior of the system was evaluated in scenarios involving clouds, cloud shadows, fog, snow/ice, and vessels; and the strengths and weaknesses of the proposed method are discussed. The results show that a large amount of data can be saved, paving the way for large reductions in transmission costs to the ground station. For the images analyzed, 77.7% of the information can be discarded without compromising the predefined useful information. The merits of the proposed method are also shown through an individual analysis of some key images belonging to the dataset. Moreover, processing time analyses were performed using Unibap’s iX10-100 space cloud platform, and we point out future directions. To the best of our knowledge, is there no other related work combining detection algorithms with one image compression algorithm aiming at remote sensing satellite data saving.

This paper is organized as follows. Section 2 describes the proposed method and the experimental framework used to evaluate its performance. We present our results in Section 3, and in Section 4 we discuss them in the larger context. Finally, Section 5 presents our conclusions.

## 2. Materials and Methods

**Figure 1:**
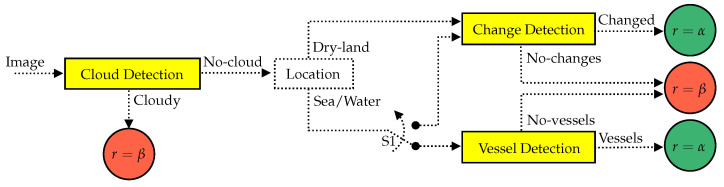
Diagram of the pre-processing step. The image (tile) compression with the coding rate α is represented by the green circles, and the compression with the coding rate β is represented by the red circles. The algorithms for cloud detection, change detection, and vessel detection are illustrated by the yellow blocks.

The proposed method relies on a pre-processing step before coding and transmitting the acquired images. The focus is on remote sensing satellite applications that agree with the following: (1) changes in dry-land regions other than clouds are considered as ROI; (2) water regions containing vessels are considered as ROI. Other content is considered as not useful. Thus, let *r* be the coding rate of the image compressor. The ROI can be compressed with a coding rate r=α, and the other content can be compressed with a coding rate r=β, for β<α. As mentioned in Section 1, the proposed method makes use of a cloud detection algorithm, a change detection algorithm, and a vessel detection algorithm. The algorithm’s workflow is shown in Figure 1.

First, the input image is segmented into tiles of δ by δ and processed by the cloud detection algorithm. The tiles classified as containing clouds (labeled as “Cloudy”) are compressed with the coding rate r=β. The other tiles (labeled as “No-cloud”) are processed by the change detection algorithm if they contain dry-land regions. The differentiation between dry-land and water regions by georeferencing is considered in this paper. For water regions, the “No-cloud” tiles can be processed by the vessel detection algorithm. Alternatively, the tiles located in water regions can be processed by the change detection algorithm, in case the target application is interested in any other changes (e.g., oil spill or icebergs) in addition to the vessels, which can also be considered as a change. In this case, the reference image used by the change detection algorithm can be, for instance, a synthetic image which best characterizes the water region or oceanic area. The option of using the change detection algorithm instead of the vessel detection algorithm is represented by the switch S1.

The ability of the change detection algorithm to detect vessels as changes must be taken into account when setting S1. The use of the vessel detection algorithm may be preferable, as cloud detection algorithms generally can not ensure a 100% detection rate. Consequently, the tiles wrongly classified as “No-clouds” will be marked as ROI by the change detection algorithm, which can reduce the performance of the system. Ideally, that problem can be solved by using the vessel detection algorithm, since detecting vessels in cloudy images should result in "No-vessels". However, the cloud detection step is still necessary, considering that the presence of clouds increases the number of false alarms produced by the vessel detection algorithm. In addition, the computational costs for vessel detection and change detection are different, varying according to the implementation of the algorithms (including optimizations) and the restrictions imposed by the platform. These issues can also be considered when setting S1 while aiming at processing time and energy savings.

Finally, the tiles containing changes or vessels are compressed with the coding rate r=α, and the other tiles are compressed with the coding rate r=β.

In this paper, baseline algorithms for the cloud detection, change detection, and vessel detection steps are proposed. The proposed method was expected to show good results, even compared with non-learning-based algorithms. However, the use of other algorithms that satisfy the constraints presented by the embedded platform can also be considered. The baseline algorithms were modeled using ForSyDe [13], which is a methodology with a formal basis for modeling and design of heterogeneous systems-on-chip and cyber-physical systems. This methodology allows modeling the baseline algorithms considering a high level of abstraction, focusing on functionality. ForSyDe envisions automated generation of optimized codes for the target platform in the near future, and a trade-off analysis of the demands and resources savings as part of the design space exploration tool in ForSyDe [14]. In this paper, Matlab and Python code was created manually based on the ForSyDe models to perform the analyses in Section 3. The baseline algorithms are detailed in the following sections.

### 2.1. Baseline Algorithm for Cloud Detection

The task of detecting clouds in satellite images is still a challenge. Different algorithms have been proposed over the years aiming at even lower FAR for the achievable values of PD. A survey of cloud detection methodologies is given in [15], in which 59 approaches are discussed among the classical and machine learning approaches.

Approaches vary in accuracy and output values. The results of classification can vary from a simple cloud/no-cloud to a wider range of labels, such as: thin cloud, thick cloud, cloud shadow, snow, ice, and others. In fact, different properties of the clouds are explored by the approaches to perform the detection and classification, which can be physical parameters (e.g., shape attributes) or optical properties (e.g., spectral content, brightness temperature, and polarization characteristics).

**Figure 2:**
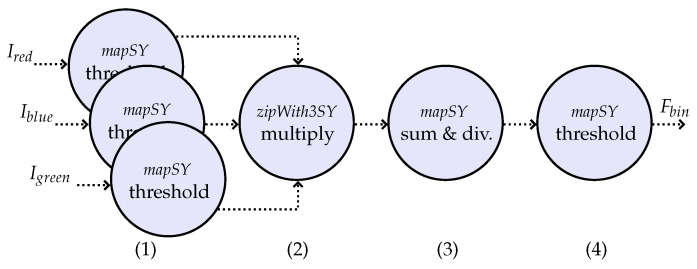
ForSyDe model for the cloud detection algorithm using the synchronous model of computation (modified from [12]). In (**1**), one threshold operation (with the value $\tau_{1}$) is applied to each one of the input bands. In (**2**), the resulting binary matrices from each band are element-wise multiplied. Then, the elements are summed and divided in (**3**) according to the dimensions of the tiles. In (**4**), another threshold operation (with the value $\tau_{2}$) is performed to label each tile as “No-cloud” or “Cloudy”.

This paper makes use of the threshold-based algorithm described in [12] to perform the detection of the clouds. More precisely, the algorithm makes use of the RGB bands as input signals to explore the brightness content, for which the threshold value may vary by season, sun elevation, and other factors, to avoid false alarms. Figure 2 shows the processing steps of the cloud detection algorithm, which can be described as follows.

First, one threshold operation (1) of value $\tau_{1}$ is applied to each one of the RGB-bands ($I_{red}$, $I_{blue}$, and $I_{green}$) of the tiled image. Next, the resulting binary matrices from each band are multiplied element-wise (2). Then, the values are summed and divided by $\delta^{2}$ (the area of the tile) (3), resulting in one value for each tile as a ratio of brightness per area. Finally, another threshold operation (4) is applied to the value calculated for each tile, with a threshold value of $\tau_{2}$, resulting in the binary flag $F_{bin}$. In fact, the last threshold operation labels each tile as “No-cloud” or “Cloudy”. The labels “mapSY” and “zipWith3SY” represent the process constructors of the ForSyDe framework [13].

### 2.2. Baseline Algorithm for Change Detection

Detecting changes between multiple images of the same scene taken at different times has a large number of applications in diverse disciplines, including remote sensing, medical, civil infrastructure, and others [16]. Typically, for remote sensing applications, this would mean either that something interesting has significantly changed in the area or that a new type of object is in an area. Several strategies can be found in the literature aiming at detecting changes in optical satellite images [17] and in synthetic aperture radar (SAR) images [18,19]. The challenge is that an image over a surveillance ground can look different every time the satellite passes, considering, for instance, the position of the sun, cloud shadows, satellite angle of incidence, satellite azimuth angle, and other factors. In urban areas, the vehicle traffic and parking lots would look different almost every time, which can be considered non-relevant content for some applications. However, there might still be interesting changes that do not involve cars, such as mass protests, fires, deforestation, and other events. In that sense, these contents can be detected by the on-board software and marked to be transmitted to the ground-station as ROI.

**Figure 3:**
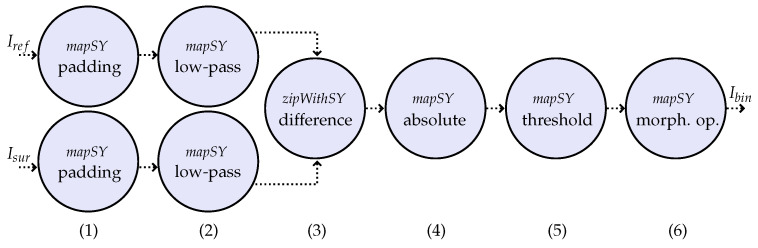
ForSyDe model for the change detection algorithm using the synchronous model of computation. In (**1**), the tiles are padded to allow the application of the low-pass filter in (**2**). Then, the resulting values of the reference image are subtracted from the values of the surveillance image in (**3**). In (**4**), the absolute values of the pixels are calculated, and in the sequence, one threshold operation is performed (with the value $\tau_{3}$) in (**5**). Morphological operations are performed in (**6**).

A comparison of representative change extraction methods based on pixel analysis is given in [17], in which different methodologies are grouped as: algebraic and statistical analysis, feature space transformation, change classification, feature clustering, and deep neural network methods. Approaches combining more than one of the representative methods can also be found. The robustness of the models was evaluated considering the detection accuracy of the changed area, and also the accuracy of the non-changed area to point out a FAR. In this paper, we propose one methodology that fits into the group of algebraic and statistical analysis to perform the change detection step. Figure 3 shows the processing steps of the change detection algorithm.

As illustrated by Figure 3, one low-pass filter is applied (2) to both the surveillance image ($I_{sur}$) and the reference image ($I_{ref}$) to reduce the influence from noise. Thus, the image (tile) is padded (1) before allowing the application of the filter to image borders. In this paper, the low-pass filter used is an averaging filter of window size 5 by 5 pixels. In the sequence, the differences in the image are computed from the averaged data by subtracting the reference image from the surveillance image (3). The absolute values of the pixels are calculated in the next step (4), since positive differences and negative differences are considered of interest. Then, the threshold operation is applied (5) to the absolute values of the image-difference. The threshold value, $\tau_{3}$, might vary according to the satellite image sensors. Overall, the choice of a reference image acquired by the same sensor as the surveillance image will avoid complications in choosing the value of $\tau_{3}$, and the use of images acquired in the same season, with same sun elevation and acquisition angle, will be beneficial. Finally, morphological operations are applied (6) to the binary output of the threshold block with the objective of reducing the false alarms related to single pixels (the circle “morph. op.”). Morphological operations can also avoid small alarming changes and deviations in georeferencing. In that sense, operations of erosion and dilation are applied. Finally, the image (tile) is labeled as containing changes if the binary output matrix $I_{bin}$ contains at least one value equal to 1.

### 2.3. Baseline Algorithm for Vessel Detection

The world merchant fleet alone counted over 99800 ships of more than 100 gross tons in 2021 [20]. This amount of vessels demands detection, classification, or identification, in satellite images, since carrying an Automatic Identification System (AIS) or Long-Range Identification and Tracking (LRIT) system is not required for vessels weighing less than 300 tons [21]. In addition, fishing vessels do not carry a Vessel Monitoring System (VMS), depending on the region. Furthermore, illegally operating vessels can spoof their mandatory position reports, and as a consequence, the cooperative systems to provide comprehensive MDA can not be considered sufficient.

An overview of 119 selected publications on vessel detection from optical satellite imagery can be found in [21]. Strategies exploit different features on images to perform the vessel detection, and their effectiveness depends on image resolution and other image properties. In [21], methodologies are classified as: threshold-based, statistical methods, transform domain methods, computer vision methods, deep learning methods, and shape and texture methods, among others.

**Figure 4:**
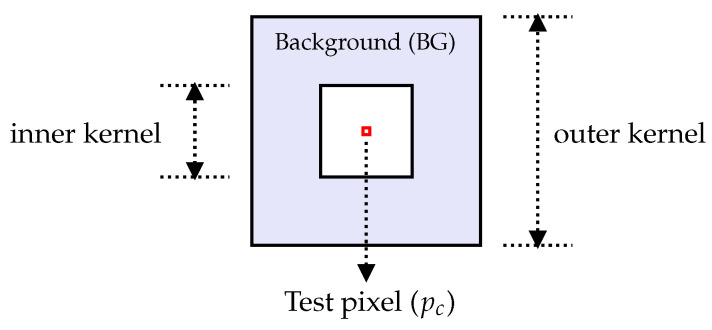
Illustration of the CFAR filter window (modified from [12]).

The threshold-based algorithm proposed in [12] was used in this study. The algorithm makes use of the constant false alarm rate (CFAR) normalization described in [22] as a part of the change detection algorithm for VHF UWB SAR images. The normalization is carried out to allow finding an appropriate threshold that can be applied globally to give a constant probability of false alarms. The normalized image is produced by centering the filter at each pixel of the image and estimating the mean and standard deviation for the pixels lying within a background window (BG), as illustrated by Figure 4.

The outer kernel and inner kernel sizes were set to, respectively, 31 by 31 pixels, and 19 by 19 pixels, as in [22]. The pixels of the output CFAR image are computed by

(1) $$p_{o}=\frac{p_{c}-mean\left(\mathrm{BG}\right)}{std(\mathrm{BG})}\;,$$

where $p_{o}$ and $p_{c}$ denote the output and central pixel values, respectively.

**Figure 5:**
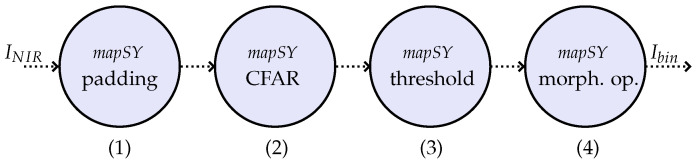
ForSyDe model for the vessel detection algorithm using the synchronous model of computation (modified from [12]). In (**1**), a padding operation is performed in the tile borders. CFAR normalization is applied in (**2**), followed by a threshold operation (with value $\tau_{4}$) in (**3**). Then, morphological operations are applied in (**4**).

The diagram containing all the processing steps of the vessel detection algorithm is shown in Figure 5. As pointed out by Figure 5, the algorithm makes use of the NIR band as input signal ($I_{NIR}$), since the reflectance of water in NIR band makes the contrast between vessels and water greater than in the other available bands [23]. First, the padding operation (1) is performed in the input image (tile) to avoid missing detection on the edges, where the amount of padded pixels relies on the size of the CFAR filter window. Then, the CFAR normalization is performed (2), followed by a threshold operation with a value $\tau_{4}$ (3). Next, similarly to what is done by the change detection algorithm, morphological operations are applied (4) to the binary output with the objective of reducing the false alarms related to single pixels. For the vessel detection algorithm, one operation of erosion, followed by one operation of dilation, are applied. Finally, the tile is labeled as containing vessels if the binary output matrix $I_{bin}$ contains at least one value equal to one.

### 2.4. Evaluation

The image compressor recommended by the Consultative Committee for Space Data Systems (CCSDS) was used to evaluate the proposed method. The CCSDS 122.0-B-2 [4] recommends a gray-scale image compressor for aerospace applications composed of a discrete wavelet transform (DWT) followed by a bit plane encoder (BPE). The implementation made available by the University of Nebraska was used to compress the tiles classified with the proposed method [24].

The CCSDS image compressor allows operating with two arithmetics: (a) integer DWT for lossless compression; (b) float DWT for lossy compression. In this study, the image compressor was set to perform the lossless compression with the coding rate (*r*) of α bits per pixel (bpp). Thus, the tiles containing the ROI can be decoded in the ground station free of distortions (regarding the source coding), but their final amount of bits is variable. In other words, compressing a tile with r=α means that α will be equal to the lowest possible value achieved by the image compressor to perform the lossless compression of the tile, which varies depending on the image pixels. This means that the α value is a variable not defined by the user.

The compressor was set to compress the tiles with losses for r=β. In this configuration, the compression rate is fixed but the distortion varies slightly depending on the image pixels. Thus, the cost in terms of amount of information is fixed for the tiles without useful content. In that sense, β=0 means that the tiles without useful content are just discarded.

Another option would be to configure the compressor to fix the distortion instead of fixing the coding rate. Then, the amount of information related to the non-relevant tiles will be variable. Both options will result in the same rate-distortion curve, since this function is intrinsic to the compressor. In this study, it was chosen to operate with a fixed coding rate to have the costs of transmitting the non-relevant content well defined.

The distortions resulting from the lossy compression were measured in terms of mean squared error (MSE), where

(2) $$\mathrm{MSE}=\frac{1}{\delta^{2}}\sum_{i=0}^{\delta -1}\sum_{j=0}^{\delta -1}{\left(p\left(i,j\right)-\hat{p}\left(i,j\right)\right)}^{2}\;.$$

p(i,j) and $\hat{p}\left(i,j\right)$ represent the pixel values of indexes i=0,⋯,δ−1 and j=0,⋯,δ−1 for the tiles, respectively, before and after the compression. In addition, values in terms of peak signal-to-noise ratio (PSNR) were calculated from the MSE values by

(3) $$\mathrm{PSNR}\;(\mathrm{dB})=10\times log_{10}\left(\frac{M^{2}}{\mathrm{MSE}}\right)\;,$$

where M denotes the maximum possible pixel value. For instance, $M=2^{16}-1=65,535$ for input images of 16 bpp.

Finally, gain in terms of information saved (*G*) was calculated as a ratio: the number of bits resulting from the application of the proposed method divided by the number of bits required for lossless compression of the whole image (usual case). Thus, the lower the gains values, the better.

### 2.5. Scenarios

Experiments were conducted considering typical scenarios containing clouds, vessels, and small changes on dry land. One area in Stockholm, Sweden, composed of both dry land and water regions, was arbitrarily selected—around the Skärpo region. The area has the following approximate central coordinates: 18.75991, 59.33307 for WGS84; and 372579, 6579199 for EPSG 32634.

Then, some images used by a Saab-developed AI-based data fusion system were selected. The images were from PlanetScope, which is a constellation of approximately 130 satellites (orbit type LEO-SSO) that is able to image the entire land surface of the Earth every day [25]. The images have approximately 3m per pixel resolution and 16 bpp precision (bit depth); they are composed of 4 bands (PSScene4Band): blue, green, red, and near-infrared bands.

Altogether, 25 images were selected and cropped to the selected area of 1300 per 1300 pixels. More precisely, the 25 images were randomly selected from those covering the selected area within the time range of approximately 8:44 to 10:10 (considering summer and winter days). The time range was limited to these values to select images with similar solar conditions, which allowed the use of a fixed threshold value for the detection algorithms during the experiments. Table 1 gives more details about the selected images.

The labels listed in the last column of Table 1 were defined after a careful, deep visual examination of the images. Then, the images were grouped as no-clouds, cloudy, small clouds, fog, snow and clouds, and snow and no-clouds. The selected images have possible vessels in the water region, excluding images 1 and 21–24, in which highlighted pixels were not detected in the water region. It is worth mentioning that the dataset is not labeled. Thus, some possible vessels could be the results of noise, movements in the water due to the wind, or very small vessels (e.g., surfboards and jet-skies). However, as the objective was to mark ROI containing possible vessels, lack of labeling was not an issue. In other words, possible vessels can be losslessly compressed in addition to real vessels to avoid loss of interesting content for the application.

**Figure 6:**
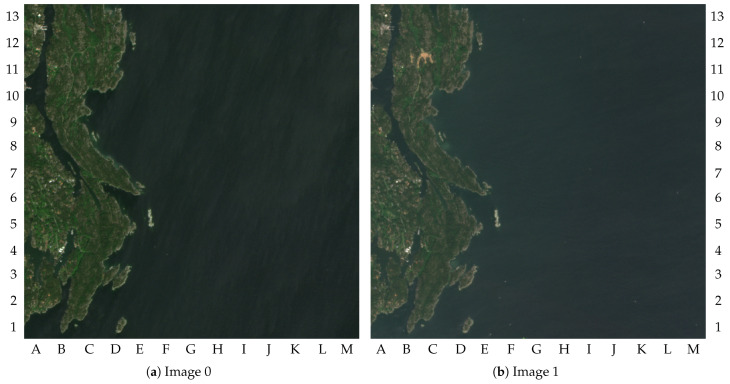
Images samples, RGB bands (truncated at 2000): (**a**) image 0; (**b**) image 1.

Figure 6 shows two images of the selected area as examples. The maximum amplitude of the pixels was truncated at 2000 to simplify the visualization of the images in this paper (i.e., the pixel values higher than 2000 are printed as being equal to 2000).

## 3. Results

The proposed system was implemented in Matlab, and the experiments were conducted using the images listed in Table 1. The parameters configured for the experiments can be summarized as follows:

- Tile size: δ=100;
- Lossy compression rate: β=0.2 bpp;
- Cloud detection threshold values: $\tau_{1}=1500$, $\tau_{2}=0.9$;
- Change detection threshold value: $\tau_{3}=300$;
- Vessel detection threshold value: $\tau_{4}=6$;
- Reference image for change detection: image 0;
- Morphological operations for vessel detection: 1 erode (length: 1 pixel), 1 dilate (length: 1 pixel);
- Morphological operations for change detection: 1 erode (length: 2 pixels), 1 dilate (length: 2 pixels).

Some comments about the parameters can also be made. For δ=100, the input images have 169 tiles. It is also worth mentioning that β equal to 0.2 was chosen empirically, as this value results in an acceptable lossy representation of the non-relevant content without requiring a large amount of data.

Furthermore, note that $\tau_{2}=0.9$ implies that at least 90% of the tiles must be covered by clouds to be labeled as “Cloudy”. Ideally, $\tau_{2}$ would be equal to one. However, parts of the clouds cannot be detected due to brightness gradients in the clouds being higher with the higher values of $\tau_{1}$. In that sense, giving a tolerance for $\tau_{2}$ can improve the performance of the detection for some values of $\tau_{1}$. Thus, both threshold values must be balanced.

Another point to be mentioned is that the threshold value for the vessel detection algorithm was empirically set to $\tau_{4}=6$, as in [12,22]. Other values for $\tau_{4}$ did not perform a better balance between PD and FAR. Gains in terms of image compression were computed using the NIR band as case of analysis. Results for the other bands can be considered analogous.

Image 0 was selected to be used as reference image by the change detection algorithm. There was no particular reason for this choice other than that image 0 had the entire dry-land region still as green, as shown by Figure 6a. Thus, the images containing changes in the green area could be detected, as illustrated by Figure 6b. The results for the 25 images are given in Table 2.

First we discuss the results for image 0. Image 0 was selected to be used as the reference image by the change detection algorithm, so the surveillance image is the same as the reference image for the results shown in the first row of Table 2. Consequently, there were no changes to be detected. The reference image does not contain well defined vessels, but three small detections were found in the water region. Two of the three detections were in the same tile. Consequently, 2 of the 169 tiles were marked as ROI by the system, and almost the whole image was lossy compressed with r=β. These detections can be due to small vessels, noise, or simply movement of water due to wind, for instance. This result can be verified in the second column of Table 2, where it can be seen that the final amount of information, measured in BPP, is close to β. The gains achieved with the proposed method are shown by columns six and seven (calculated as defined in Section 2). To facilitate further comparisons, columns eight and nine show the BPP values achieved in the case of lossless compressing the images with the CCSDS image compressor and with JPEG2000—using the implementation provided by OpenJPEG [26]—respectively.

**Figure 7:**
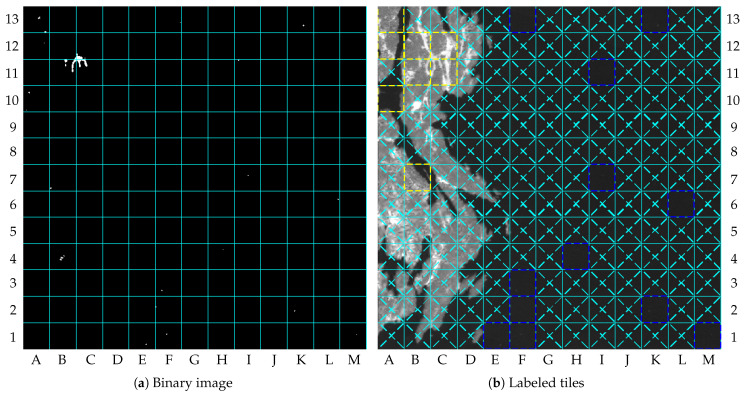
Results of image 1: (**a**) binary image; (**b**) labeled tiles (truncated at 4000).

The result of image 1 describes a scenario without clouds containing changes in the dry-land region, as illustrated by Figure 6b. The major changes in dry-land regions can be observed by comparing the upper-left quadrants of Figure 6a,b. Figure 7a shows the combined binary images resulting from the cloud detection algorithm, change detection algorithm, and vessel detection algorithm. The final labeled tiles are illustrated in Figure 7b. In this paper, the labeled tiles are marked as follows:

- Tiles with ROI in dry-land regions are outlined with yellow traced lines;
- Tiles with possible vessels in water region are outlined with blue traced lines;
- Cloudy tiles are crossed with red traced lines.
- Other tiles without interesting content are crossed with cyan traced lines;

Figure 7b shows the NIR band of image 1 compressed with the proposed method, in which the labeled tiles are marked. Tiles A10, A12, A13, B7, B11, B12, C11, and C12 were labeled as containing changes, whereas tiles E1, F1, F2, F3, F13, H4, I7, I11, K2, K13, L6, and M1 were labeled as containing vessels. The other remaining tiles were labeled as not containing interesting content.

**Figure 8:**
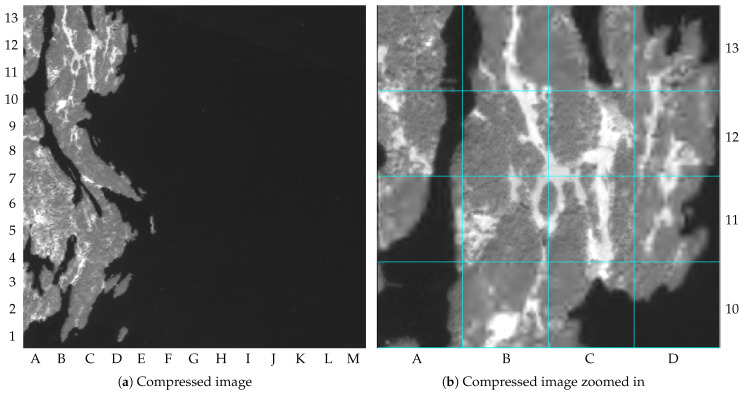
NIR band of the image 1 compressed with the proposed method (truncated at 4000): (**a**) compressed image; (**b**) compressed image zoomed in.

As pointed out by Table 2, the image distortion achieved for image 1 was 59.5 dB of PSNR. To simplify observing the image distortion’s impacts, Figure 8a shows the NIR band compressed with the proposed method but without the tile markings, and Figure 8b shows its upper-left quadrant zoomed in. The differences in image distortion between the tiles compressed with r=α and r=β can be seen, for instance, when comparing the tiles B13 and C13 (lossy compressed) with the tiles B12 and C12 (lossless compressed). It is important to mention that the images in this paper contain small amounts of distortion resulting from the editorial process, such as resizing or file manipulation.

The final amount of information required to compress the NIR band of the image 1 is 1.09 bpp, which results in gains of 0.155 for β=0.2, and 0.128 for β=0. This means that only 15.5% of the information required for lossless compression of the whole image is needed. This percentage can be reduced to 12.8% in case of discarding the tiles without ROI.

**Figure 9:**
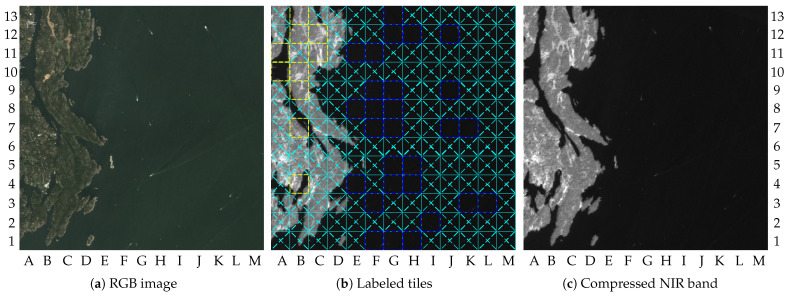
Results for image 2: (**a**) RGB image (truncated at 2000); (**b**) labeled tiles (truncated at 4000); (**c**) compressed NIR band (truncated at 4000).

Image 2 results in smaller gains. It requires 26.3% and 23.6% of the information, respectively, for β=0.2 and β=0. The differences in the gains between image 1 and 2 are due to the numbers of tiles marked as ROI. Indeed, 38 tiles were marked as ROI for image 2, which is almost the double the number for image 1. As a consequence, the increase in the number of tiles that were losslessly compressed raised the final coding rate to 1.67 bpp. An increase in image quality was achieved also, resulting in a PSNR value of 60.3 dB. Figure 9a–c show the RGB image, the labeled tiles, and the compressed NIR band image, respectively.

As shown by Figure 9a, image 2 has new changes in the upper-left quadrant of the image and many vessels in the water regions. The changes in dry-land region comprise 10 tiles, and possible vessels cover 28 tiles, as illustrated by Figure 9b. According to Table 2, the same number of tiles marked as ROI in image 2 was marked in images 14 and 17, but resulting in different values of gains. As mentioned in Section 2, the coding rate α is variable in order to ensure lossless compression. For the lossily compressed tiles, β was fixed to 0.2, which generated the differences in the PSNR values. Thus, the differences in gains for images 2, 14, and 17, were due to the lossless compression. The same was true for other images with the same number of ROI.

The results for most other no-cloud images in Table 2 are similar. Variations were observed both in the number of tiles marked as containing changes on dry land and in the number of tiles marked as containing vessels in water regions, resulting in gains between 0.155 and 0.263 for β=0.2. The exception is observed for image 8, in which 56 tiles were marked as ROI: 35 tiles were marked as containing changes in dry land and 21 tiles were marked as containing vessels in water regions. The increase in the number of tiles marked in dry land was a result of deviations between the surveillance image and the reference image due to image corrections and calibrations. In fact, the results for image 8 illustrate possible consequences of deviations in georeferencing, differences in sensors calibrations, or failure of other corrections to the images.

**Figure 10:**
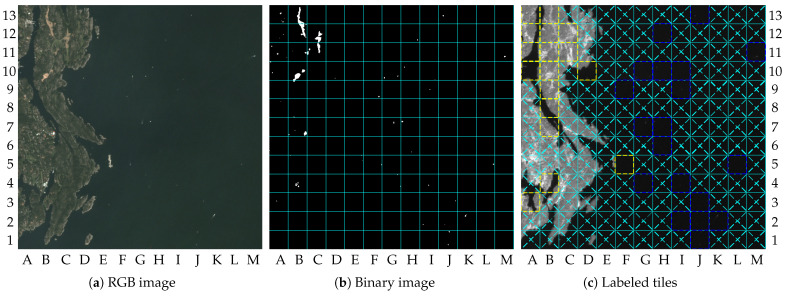
Results for image 5: (**a**) RGB image (truncated at 2000); (**b**) binary image; (**c**) labeled tiles (truncated at 4000).

Another point that can be observed concerns the border between dry-land and water regions. In this paper, the tiles containing partially dry land and partially water regions were marked as dry land. In turn, such tiles are processed by the change detection algorithm (i.e., not by the vessel detection algorithm). This choice was made because the change detection algorithm can detect some vessels in the water region as changes if the reference image does not contain the same vessel in the same position, while the vessel detection algorithm is not able to detect changes in the dry-land region. In a real application in the future, the proposed method can be adjusted to allow the execution of both algorithms if the application requires more accurate vessel detection in these tiles. The results of image 5 are brought by Figure 10 and can be used to illustrate that issue.

Figure 10a shows image 5, in which many vessels can be observed in the water region. One of them is located near a small island around the tile F5, which was defined as dry land because it contains part of the island. Figure 10b shows detection in tile F5, close to the tile G5, and as a consequence, tile F5 was labeled as containing changes by the system, as shown by Figure 10c.

**Figure 11:**
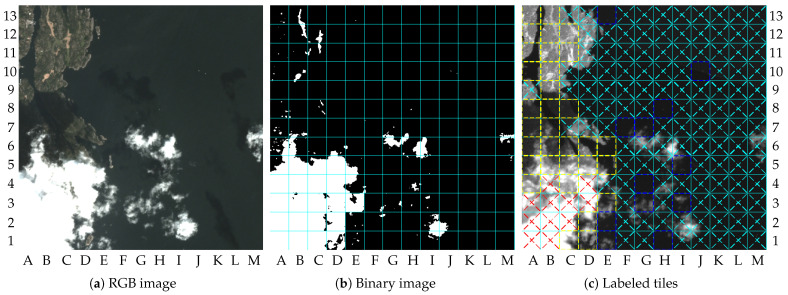
Results for image 9: (**a**) RGB image (truncated at 2000); (**b**) binary image; (**c**) labeled tiles (truncated at 4000).

The system’s behavior in scenarios containing clouds can also be discussed. Initially, some comments can be made by analyzing the results achieved with image 9. Figure 11a shows the RGB-bands image of image 9, where it is possible to observe the cloud portion mostly in the lower-left quadrant. In addition, small portions of clouds can be found over the water region. The shadows of the clouds on the ground can be observed in both dry-land and water regions.

Figure 11c shows the tiles labeled by the system. Tiles labeled as “Cloudy” can be found in the lower-left quadrant of Figure 11c. This is the case for tiles A1, B1, A2, B2, C2, A3, B3, C3, D3, B4, and D4. The other tiles with clouds in dry land were wrongly labeled as containing changes. The reason for this is: (1) some parts of the clouds into the tiles are not sufficiently bright to cross the threshold $\tau_{1}$ (e.g., tile A4), or (2) the tiles are not sufficiently covered by clouds to cross the threshold $\tau_{2}$ (e.g., tile B6). As mentioned in Section 2, the tiles wrongly classified as “No-clouds” can be marked as ROI by the change detection algorithm. The real changes detected in dry land are similar to those detected in image 5, which are located at the upper-left quadrant. In addition, tiles labeled as containing changes due to the shadows of the clouds can also be observed. This is the case for tiles A8, B8, and C8, for instance. Cloud shadows can generate differences between the image and the reference image that exceed the threshold value $\tau_{3}$, as pointed out by the white dots in the tiles A8, B8, and C8, of Figure 11b. In general, detection failures caused by cloud shadows, or by the undetected clouds, will reduce the system’s performance on dry land. In water regions, false alarms caused by cloud shadows were not observed. However, false alarms on the vessel detection step can be seen due to small portions of clouds, which is the case for tiles F7, G7, I5, G4, G3, I3, E1, and H1. Tiles E2, H8, J10, and E13, can be considered tiles containing well-defined small vessels.

**Figure 12:**
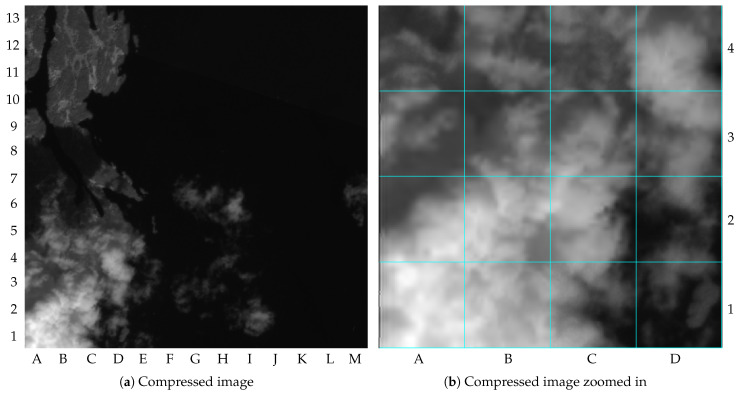
NIR band of the image 9 compressed with the proposed method: (**a**) compressed image; (**b**) compressed image zoomed in.

In fact, false alarms caused by undetected clouds can reduce the efficiency of the proposed method in terms of information saved, but they cannot compromise the useful information. To illustrate that issue, Figure 12a shows the NIR band of the image 9 compressed with the proposed method, and Figure 12b shows its lower-left quadrant zoomed in. The tiles A4, C4, D2, D1, and C1, were lossless compressed, but the other tiles in Figure 12b were lossy compressed. Thus, uninteresting content (e.g., tile A1) had loss of details, and content with uncertainty had its details preserved (e.g., tile A4). According to Table 2, the PSNR value and the final coding rate reached for image 9 were, respectively, 63.3 dB and 2 bpp. The gains achieved by using the proposed method were 0.320 and 0.294 for β=0.2 and β=0, respectively.

**Figure 13:**
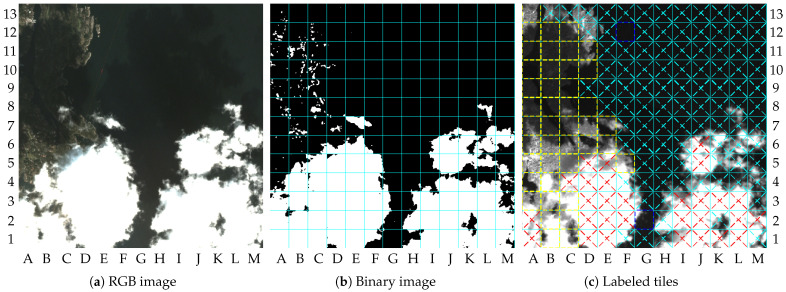
Results for image 10: (**a**) RGB image (truncated at 2000); (**b**) binary image; (**c**) labeled tiles (truncated at 4000).

Figure 13 shows the results of the proposed method applied to the image 10, which is another cloudy image. Similarly to the results of image 9, cloud shadows on the ground can be seen to increase the number of tiles marked as “changed” in the dry-land region. At all, the tiles classified as “No-changes” were the tiles B13, C13, D13, D12, and E12. Other tiles that were lossily compressed and showed dry land were due to the presence of clouds itself (cloudy tiles), such as tiles A1, A2, and C4, for instance. In the water region, two tiles were wrongly marked as containing vessels. Tile F12 resulted in a false alarm due to noise in image synthesis, and tile G2 resulted in a false alarm due to the presence of clouds. The other tiles in the water region were not classified as ROI, and were compressed with the coding rate β. The gains for image 10 were 0.307 for β=0.2 and 0.283 for β=0.

**Figure 14:**
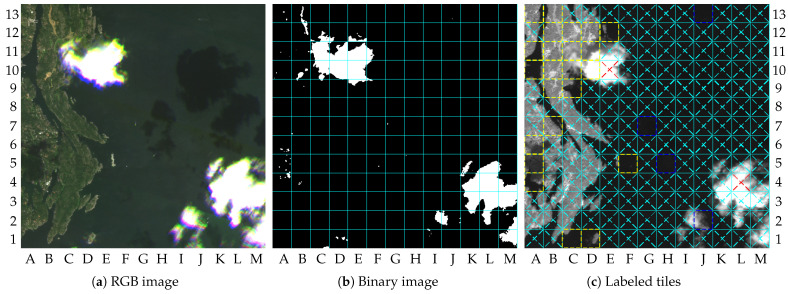
Results for image 12: (**a**) RGB image (truncated at 2000); (**b**) binary image; (**c**) labeled tiles (truncated at 4000).

The cloud cover in image 11 is similar to that in image 10, producing somewhat similar gains, as described in Table 2. In image 12, the cloud portion is slightly smaller than the cloud portion in image 10, as shown by Figure 14a. Thus, the negative impacts of cloud shadows on the change detection step are smaller, being limited to tiles A4, A5, and A6, as illustrated by the detections in Figure 14b.

In the water region, four tiles were correctly marked as containing vessels. In particular, it is interesting to observe tile J2 in Figure 14c, which contains two possible vessels almost covered by clouds (near the border with tile K2). Tile J2 is an example of a tile partially covered by clouds, but with interesting content elsewhere. This justifies using high values of $\tau_{2}$ to avoid missing out on interesting content.

**Figure 15:**
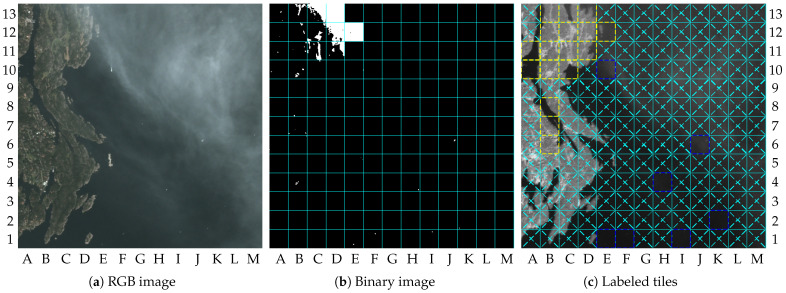
Results for image 18: (**a**) RGB image (truncated at 2000); (**b**) binary image; (**c**) labeled tiles (truncated at 4000).

**Figure 16:**
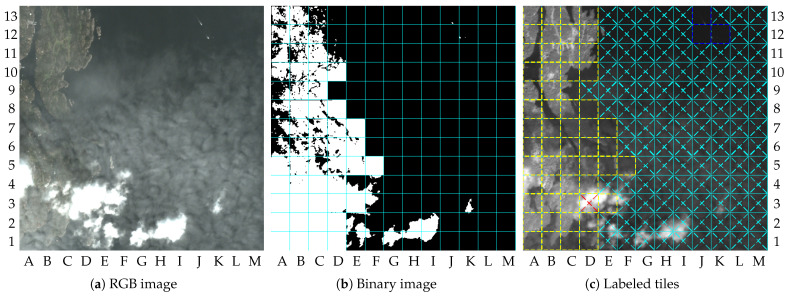
Results for image 20: (**a**) RGB image (truncated at 2000); (**b**) binary image; (**c**) labeled tiles (truncated at 4000).

The results of images 13 to 16 are similar to those already discussed for the group of images containing clouds. Moving forward with the description of the results, the behavior of the system in fog scenarios can be discussed through the results of images 18 and 20. Figure 15a illustrates a case of moderate fog, whereas Figure 16a is a scenario of denser fog along with small clouds.

Most of the fog is over the water region in image 18. Thus, the fog is not affecting much of the change detection in the dry-land region. The damages are basically in tiles C13, D13, D12, E12, and D11, where the differences from the reference image exceed the threshold $\tau_{3}$. The other tiles marked as ROI on dry land can be considered the result of very small and punctual changes. Differently, the fog greatly compromised the detection of changes in image 20, in which almost all the tiles were marked as ROI in Figure 16c. The exception are tiles E12 and D3, the latter being eliminated by the cloud detection algorithm.

For the water region, the results of image 18 reveal that most of the vessels were detected even with the fog. The tiles containing vessels but not marked as ROI exist because fog reduces the contrast between the water and the vessel, changing the pixels’ statistic in the CFAR normalization filter window. This was the case for tiles M6 and G7, for instance. Thus, some pixels related to small vessels ended up not reaching the threshold $\tau_{4}$. In the case of image 20, all vessels that could be perceived were detected. As shown by Figure 16a, two vessels can be seen at the top-right quadrant, resulting in three tiles marked as ROI in Figure 16c, as one of them is located on the boundary between tiles J12 and K12.

**Figure 17:**
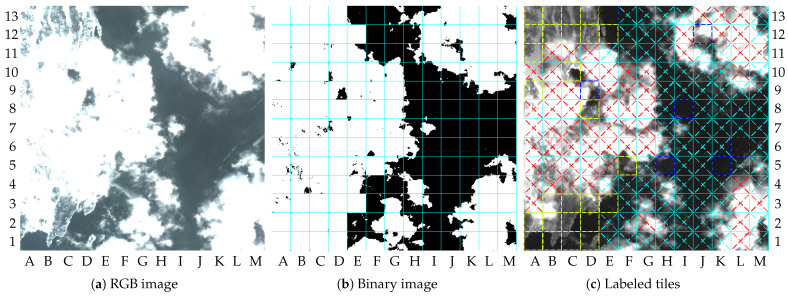
Results for image 21: (**a**) RGB image (truncated at 2000); (**b**) binary image; (**c**) labeled tiles (truncated at 4000).

The behavior of the proposed method with images containing snow and ice can be initially discussed through Figure 17, which shows the results of image 21. Image 21 is a scenario with both snow and clouds. Figure 17a shows the RGB-bands image of image 21 with the maximum value truncated at 2000, in which the bright pixels resulting from both snow and clouds can be observed. Considering that $\tau_{1}=1500$, detections from both sources can be equally perceived by the cloud detection algorithm. However, the dry-land tiles were not wrongly classified as cloudy, as shown by Figure 17c. That is because in the case of image 21, the volume of snow in the area was not sufficient to exceed threshold $\tau_{2}$ due to vegetation and terrain topography. The tiles of dry-land region that are not covered by clouds were marked as ROI by the change detection algorithm. Figure 17b shows that all the pixels of the tiles processed by the change detection algorithm were marked because they reached threshold $\tau_{3}$. In the water region, the vessel detection algorithm produced seven false alarms due to clouds. Overall, the gains for image 21 were 0.237 for β=0.2 and 0.205 for β=0.

The gains achieved with image 22 were 0.372 for β=0.2 and 0.351 for β=0, which are less attractive than those achieved with image 21. The amount of snow in image 22 is less than that in image 21, but also distributed over the entire dry-land area. The difference in the gain values of the two images occurred because almost all tiles were classified as containing changes in image 22, as there were no clouds over the dry-land region. The other characteristics presented by both images are similar.

**Figure 18:**
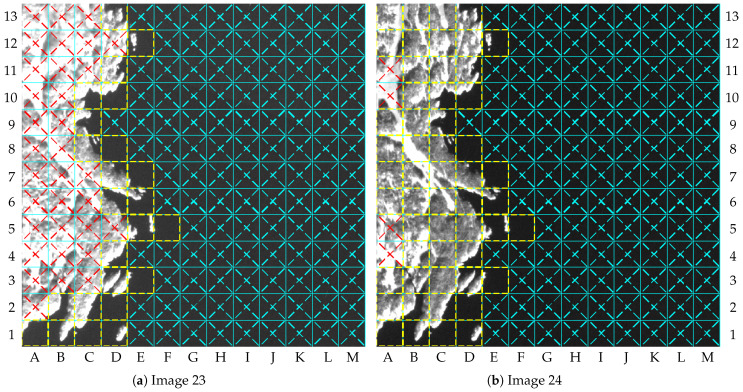
Labeled tiles of images 23 and 24 (NIR band truncated at 4000): (**a**) image 23; (**b**) image 24.

The results for images 23 and 24 can be used to describe some undesirable outcomes. Figure 18 shows the labeled tiles for both images. Differently from image 21, image 23 has no clouds, but 33 tiles in dry-land region were marked as “Cloudy”, as shown by Figure 18a. This is because both $\tau_{1}$ and $\tau_{2}$ were reached due to snow. As a consequence, most tiles with snow were lossily compressed, which may be considered undesirable for some applications. For image 24, four tiles were wrongly lossily compressed on dry land due to snow: tiles A4, A5, A10, and A11. In turn, the gains of image 23 are better than those of image 24; however, the distortions of image 23 are greater.

## 4. Discussion

**Figure 19:**
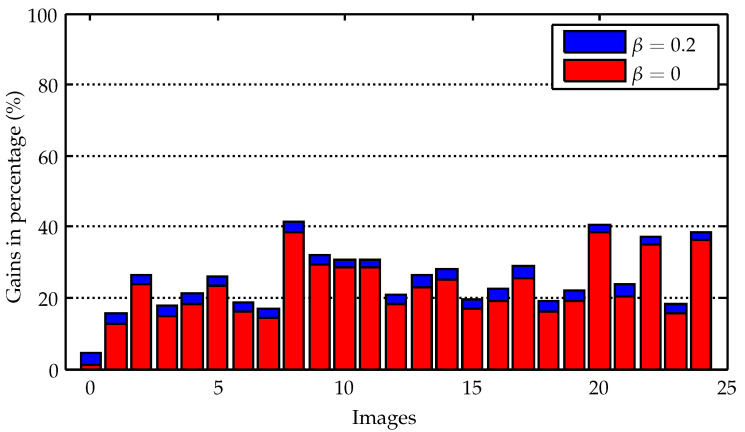
Percentage gains for the evaluated images.

The average gains for the 25 images were 0.251 (25%) for β=0.2 and 0.223 (22.3%) for β=0. The individual gains in terms of percentage achieved for the 25 images with the application of the proposed method can be compared in Figure 19.

The best gains observed were for image 0, reaching around 4.3% for β=0.2 and 1.1% for β=0.2. As mentioned in Section 3, image 0 is a case of no changes in the dry-land region and two tiles containing possible vessels in the water region. That result can be considered compatible with scenarios of entirely images of water regions containing few vessels.

If all the tiles of image 0 are not marked as ROI, and consequently the whole image 0 is lossy compressed with encoding rate β=0.2, the gain would be 0.030. This is around 3% of the information needed by the whole losslessly compressed image. This can be considered the case for ocean images without vessels, for instance. On the other hand, the images will be entirely losslessly compressed in dry-land areas, having changes in all of their tiles, which indicates that no information will be saved, resulting in gains equal to 1. The same goes for images entirely in water regions containing vessels in all of their tiles.

**Figure 20:**
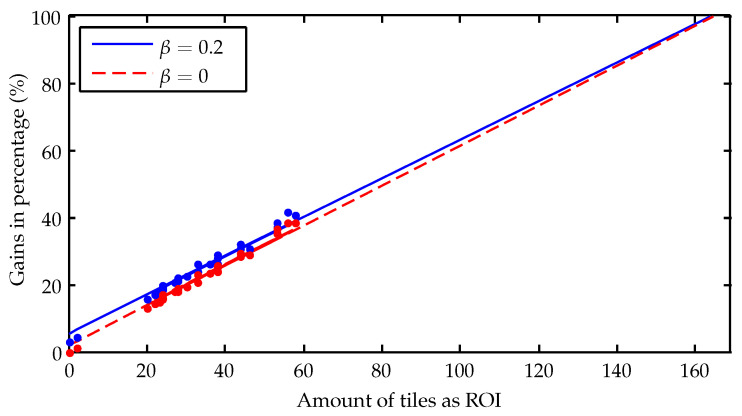
Percentage gains by number of tiles classified as ROI.

In this sense, from 2 to 58 tiles were marked as ROI for the images evaluated, within a range of possible amount of tiles from 0 to a maximum of 169. Estimates of gains for the other possible number of tiles can be performed through a linear curve fitting. Figure 20 shows the estimated curves of gains for β=0.2 and β=0, together with the gains achieved for each image (the dots round its respective curves). The behavior of both curves needs to be the same for 169 tiles, as there is no information being saved in this situation (i.e., gains of 100%). On the other hand, when the number of tiles as ROI is equal to zero, the gain for β=0 is also zero, indicating that all information is being discarded. For β=0, zero tiles means that all the tiles are lossily compressed, so the gain must be greater than zero. The curves should better fit this theoretical behavior for a larger number of images.

To discuss the weaknesses of the proposed method, the following needs to be considered. In general, detection algorithms present a trade-off between PD and FAR, in which maximizing the PD generally implies an increase in the number of false alarms. This can be considered a multi-objective optimization problem. Thus, the application rules the balancing point between the two. For the proposed method described in this paper, the policy is to ensure the transmission of all important content. Consequently, the PD of the change detection algorithm should be as high as possible, since false alarms on change detection results in unimportant content marked as ROI, and not the opposite. The same is true for the vessel detection algorithm. Thus, the parameters of both algorithms were chosen to prioritize the PD. However, the same is not valid for the cloud detection algorithm. False alarms can cause loss of important content (e.g., a tile containing snow), whereas an undetected cloud can lead unimportant content to be marked as ROI. In turn, the choice of parameters for the cloud detection algorithm should prioritize the minimization of false alarms, even if this leads to a decrease in the PD.

As discussed in [27], cloud detection is a challenge for cloud and snow coexisting areas, since both have similar spectral characteristics in the visible spectrum. To overcome this challenge, some methodologies based on machine learning have been proposed. Most of them focus on exploring textures and other image features to improve the accuracy of automated methods. However, some machine learning models for cloud detection are large, which limits their applicability and explainability. Thus, the use of these models has not been investigated at this time, but may be carried out in the future.

In general, the gains on dry-land regions are expected to be similar to or better than the gains of the global cloud fraction over dry land, which were 55%, even considering lack of detections in the cloud detection step. This expectation is considering that several tiles in dry-land regions over the globe can be classified as not containing changes. Actually, a cloud is a change, but an undesirable one for the application. With respect to the water regions, the proposed method can significantly reduce the transmission costs for applications which rely on vessel detection. In addition, the number of false alarms due to undetected clouds are lower for the vessel detection algorithm when compared to the change detection algorithm.

Finally, a short discussion about the processing time of the algorithms can be given.

### Processing Time Analyses

Processing time experiments were conducted in the Unibap’s flight representative hardware for the Unibap iX10-100 space cloud hardware solution (iX10-ADS) [28]. iX10-100 is based on the 14 nm AMD Ryzen V1000 family. It offers up to 8 processor threads and up to 8 CU graphics acceleration units, along with a PCI express generation 3.

The baseline algorithms for cloud detection, change detection, and vessel detection were implemented in Python to be evaluated separately using a single core. Processing times measured are as follows:

- Cloud detection algorithm: 0.47 s (image 11);
- Change detection algorithm: 0.47 s (image 1);
- Vessel detection algorithm: 88.48 s (image 2).

The processing time required by the cloud detection and change detection algorithms fits into a small window of time between image acquisitions. However, the processing time for the vessel detection algorithm can be considered not suitable. The large amount of processing time for the vessel detection algorithm is due to the CFAR normalization. The CFAR normalization computes each pixel of the image sequentially, since it was implemented to run in a single core. Thus, an optimized implementation aiming at parallel pixel processing is expected to considerably reduce processing time, for instance, by making use of available GPU cores.

## 5. Conclusions

This paper describes a methodology that aims at significant data saving in the transmission of remote sensing satellite images by proposing an on-the-fly analysis to decide which information is effectively useful for specific target applications before compressing and transmitting. As a consequence, bandwidth reduction can be achieved. The proposed system classifies the content as important or not important in order to define regions of interest. Then, the image compressor recommended by the CCSDS is used to compress the ROI with a coding rate α, and the non-important content with a coding rate β. To make it possible, the proposed method relies on a cloud detection algorithm, a change detection algorithm, and a vessel detection algorithm. Baseline algorithms for these three detections steps were proposed and formally modeled considering the ForSyDe framework. Then, the algorithms were implemented in Matlab and integrated according to the proposed method to simulate the compression gains. The algorithms were also implemented in Python in order to be executed on the satellite platform iX10-100, aiming at measuring their processing times.

Experiments were conducted using 25 images from PlanetScope cropped to a selected area of the Stockholm archipelago, Sweden. The images were selected in order to test the behavior of the system in different situations in both dry-land and water regions. The analyses involved scenarios containing: clouds, cloud shadows, fog, snow/ice, and vessels. Thus, one can evaluate the behavior of the system and observe the strengths and weaknesses of the proposed method.

The average gain achieved in case of lossy compressing the regions other than ROI was 0.251, which means that the information to be transmitted was reduced to around 25% of the information required to transmit the images just losslessly compressed. Individually, the gains of each image were in the range of 4.3% to 40.6%. Small improvements to the gains were reached when discarding the regions other than ROI, taking the range of gains to 1.1% to 38.4%, and the average gain to 22.3%. This amount of data saving illustrates the merits of our proposal. We expect that future low-orbit imaging satellites with limited data transmission capacity for specific missions can benefit from this study. The proposed method can also be used in multispectral satellites that work with a larger number of available bands (e.g., PlanetScope SuperDove). In this case, we recommend using the same bands (or bands of similar wavelength) as input to the detection algorithms. Experiments with images from other bands can be carried out in future works.

A few weaknesses of the proposed method were observed and discussed, pointing out future directions for the proposed system. First, one can mention the false alarms for cloud detection due to snow/ice on the ground results in lossy compression of the misclassified tiles. To overcome this challenge, the use of more robust cloud detection methodologies can be investigated. Other false alarms observed impact the information savings, but do not compromise the information considered useful.

Finally, the need for improvements in the processing time of the vessel detection algorithm can also be mentioned. An implementation aiming at the parallel processing of image pixels in the CFAR normalization step must be performed. In that sense, the automated generation of optimized codes for the target platforms can be pursued considering the design space exploration tools envisioned by ForSyDe. Reductions in processing time can also lead to reductions in energy consumption, since the runtime is an important component in the energy consumption of the embedded platform [2]. Generally, energy consumption measurement is hardware-dependent and relies on how the algorithms are implemented, depending on whether the implementation makes use of parallel processing, GPU processing, and other features. We expect that the energy savings in data transmission will be greater than the energy required to make use of the proposed method, resulting in benefits that go beyond bandwidth reductions and lower transmission costs. Analyses involving energy consumption are not within the scope of this paper, but can be carried out in future work.

## Figures and Tables

**Table 1:** Images details.

Image	Date	Time (UTC)	Platform	Instrument	Details
0	2020-06-10	10:07:59	1061	PS2.SD	no-clouds
1	2020-06-20	10:09:24	1066	PS2.SD	no-clouds
2	2020-07-17	09:42:17	1040	PS2	no-clouds
3	2020-06-14	09:41:23	1003	PS2	no-clouds
4	2020-06-27	09:41:43	1027	PS2	no-clouds
5	2020-07-17	09:36:55	1034	PS2	no-clouds
6	2020-07-20	08:44:31	106d	PS2.SD	no-clouds
7	2020-07-24	08:45:15	106d	PS2.SD	no-clouds
8	2020-08-16	09:14:40	2235	PSB.SD	no-clouds
9	2020-07-12	09:37:36	1005	PS2	clouds
10	2020-08-25	09:43:03	1034	PS2	clouds, no-vessels
11	2020-07-27	10:06:39	105a	PS2.SD	clouds
12	2020-07-24	10:09:45	1060	PS2.SD	clouds
13	2020-07-09	09:13:40	2259	PSB.SD	small clouds
14	2020-07-22	09:13:40	2277	PSB.SD	small clouds
15	2020-08-17	09:45:32	1026	PS2	small clouds
16	2020-08-21	09:12:56	222b	PSB.SD	small clouds
17	2020-07-20	09:11:52	2235	PSB.SD	fog
18	2020-08-07	09:39:37	0f17	PS2	fog
19	2020-08-07	09:37:09	0f15	PS2	fog
20	2020-08-30	09:39:13	0f34	PS2	fog
21	2018-03-16	09:24:33	0e16	PS2	snow, clouds, no-vessels
22	2019-02-20	09:34:19	1042	PS2	snow, clouds, no-vessels
23	2019-02-04	09:35:57	1018	PS2	snow, no-clouds, no-vessels
24	2019-02-06	09:30:34	1010	PS2	snow, no-clouds, no-vessels

**Table 2:** Numerical results.

Image	Bits per Pixel	Mean Squared Error	Peak Signal-to-Noise Ratio	Amount of ROI Tiles	Gain for β=0.2	Gain for β=0	Bits per Pixel with CCSDS	Bits per Pixel with JPEG2000
0	0.29	5896.2	58.6	2	0.043	0.011	6.66	6.56
1	1.09	4834.0	59.5	20	0.155	0.128	7.02	6.93
2	1.67	4006.4	60.3	38	0.263	0.236	6.37	6.27
3	1.07	3701.5	60.6	23	0.177	0.146	6.06	5.95
4	1.29	2976.4	61.6	28	0.210	0.181	6.14	6.02
5	1.66	3069.4	61.4	36	0.261	0.234	6.35	6.25
6	1.31	5701.3	58.8	24	0.187	0.160	7.04	6.95
7	1.21	4668.8	59.6	22	0.170	0.143	7.10	7.01
8	1.93	449.4	69.8	56	0.414	0.383	4.65	4.58
9	2.00	1984.6	63.3	44	0.320	0.294	6.24	6.15
10	2.07	4247.0	60.0	44	0.307	0.283	6.73	6.67
11	2.22	2883.9	61.7	46	0.309	0.287	7.20	7.14
12	1.43	3916.0	60.4	27	0.207	0.180	6.93	6.84
13	1.33	1462.8	64.7	33	0.263	0.228	5.07	5.02
14	1.53	1993.4	63.3	38	0.282	0.251	5.44	5.40
15	1.29	2737.8	61.9	24	0.197	0.169	6.53	6.44
16	1.20	2042.5	63.2	30	0.224	0.191	5.36	5.31
17	1.47	1214.6	65.5	38	0.289	0.256	5.08	5.01
18	1.22	3067.9	61.5	24	0.191	0.162	6.37	6.26
19	1.39	2612.4	62.1	28	0.219	0.191	6.36	6.26
20	2.62	390.9	70.4	58	0.406	0.384	6.45	6.35
21	1.30	5848.8	58.6	33	0.237	0.205	5.47	5.58
22	2.60	1139.6	65.8	53	0.372	0.351	6.99	6.92
23	1.38	19,384.8	53.4	24	0.182	0.158	7.58	7.53
24	2.83	4001.6	60.3	53	0.385	0.365	7.37	7.31

## Data Availability

This study made use of 25 images (cropped) from PlanetScope for the simulations, as described in Section 2. More information about the PlanetScope images can be found in [25].
